# When Seizures Lead to the Incidental Finding of Silicone Oil Migration

**DOI:** 10.7759/cureus.78633

**Published:** 2025-02-06

**Authors:** Tiago Ferreira, Angelo Dias, Sandra D. Rebelo, Ana Tornada

**Affiliations:** 1 Internal Medicine, Hospital de Santa Maria, Unidade Local de Saúde de Santa Maria, Lisboa, PRT; 2 Radiology, Hospital de Santa Maria, Unidade Local de Saúde de Santa Maria, Lisboa, PRT; 3 Internal Medicine, Hospital Professor Doutor Fernando Fonseca, Unidade Local de Saúde Amadora/Sintra, Lisboa, PRT; 4 Internal Medicine, Faculdade de Medicina, Universidade de Lisboa, Centro Académico de Medicina de Lisboa, Lisboa, PRT

**Keywords:** cerebral ventricle, imaging findings, post vitreoretinal surgery, silicone oil, silicone oil migration

## Abstract

Silicone oil, commonly used as a temporary intraocular tamponade in retinal detachment repair, may have retrobulbar migration as a possible complication of the procedure to the cerebral ventricles.

This report presents the case of a 73-year-old male patient with multiple cardiovascular risk factors and diffuse atherosclerotic disease who underwent vitrectomy of the left eye several years earlier due to retinopathy and was diagnosed through retrospective analysis of all cerebral imaging studies, with the migration of silicone oil particles, from the vitreous humor of the eye to the intraventricular space. He was admitted due to transient altered consciousness in the context of a probable seizure, with acute hyperdensity in the temporal horn of the left lateral ventricle observed in the cranial computed tomography (CT), suggestive of hemorrhage, along with signs of prior hemorrhage in the frontal horn of the same ventricle. The follow-up CT showed reabsorption of the hyperdensity seen in the admission CT, with a new hyperdense focus observed in the frontal horn of the same ventricle, a location matching that was seen in imaging studies performed eight years earlier. This focus exhibited a similar morphology, consistent with the mobilization of hyperdensity particles, according to the patient’s head movements, which, considering the patient's medical history, led to the diagnosis of intraventricular migration of the silicone oil used in tamponade of the retinal detachment repair.

The aim of this report is to describe a rare occurrence of intraocular silicone oil migration into the cerebral ventricles, which may be misinterpreted as intraventricular hemorrhage. This case highlights the importance of obtaining a detailed medical history with an integrative perspective and underscores the value of interdisciplinary collaboration in the diagnostic process.

## Introduction

Silicone oil was initially introduced by Cibis in 1962 as a temporary intraocular tamponade for managing retinal detachment associated with proliferative vitreoretinopathy. Since then, its application has broadened to include cases of complex retinal detachments and retinal tears [1,2]. This material is very stable, nontoxic, and remains insoluble in body fluids, characterized by high surface tension and significant interfacial tension with water. Following anatomical success, the removal of silicone oil is generally advised, as its prolonged presence in the vitreous cavity can result in various complications. Complications associated with the procedure include silicone oil migration into the anterior chamber of the eye, accumulation in the periorbital region, and, in rare cases, migration into the subconjunctival space or cerebral ventricular system [3]. Emulsification of silicone oil has been recognized as a common cause of many silicone oil-related complications [4].

Silicone oil migration is an uncommon occurrence, with only a limited number of case reports documenting its migration into the cerebral ventricles [5]. Diagnosis typically relies on observing the movement patterns of silicone particles in imaging exams and identifying a characteristic chemical shift artifact on brain magnetic resonance imaging (MRI) [6]. We present a fortuitous case of intraventricular migration of silicone oil, which mimicked intraventricular hemorrhage on CT imaging in a patient undergoing antithrombotic therapy.

## Case presentation

This report presents the case of a 73-year-old male patient with insulin-treated type 2 diabetes, hypertension, dyslipidemia, and diffuse atherosclerotic disease resulting in cerebrovascular disease, ischemic coronary disease, chronic kidney disease (stage IV), retinopathy, and glaucoma. The patient had also undergone a vitrectomy of the left eye 12 years prior to admission due to advanced diabetic eye disease complicated by amaurosis.

**Figure 1:**
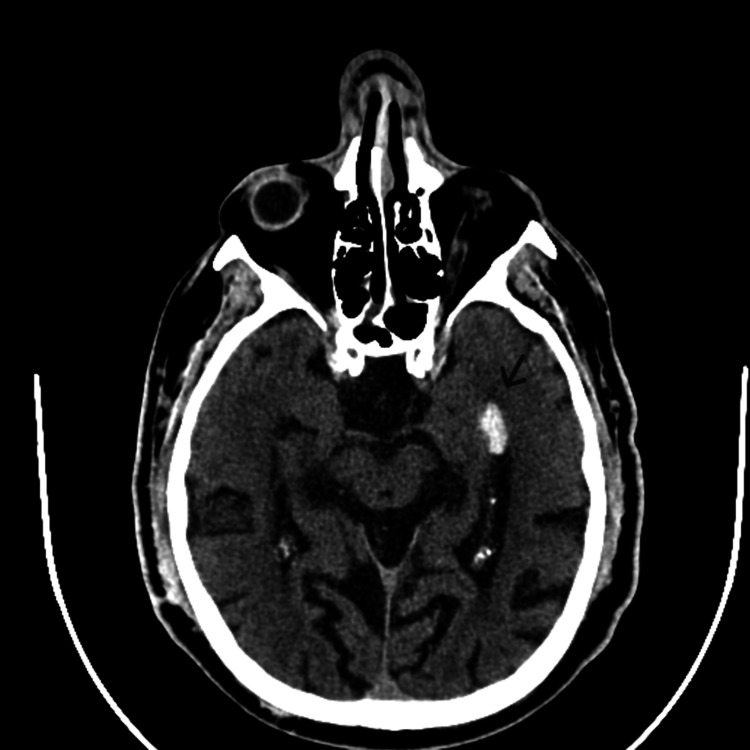
Non-contrast-enhanced brain CT showing a well-defined and rounded acute hyperdensity in the temporal horn of the left lateral ventricle (arrow)

He was admitted due to altered consciousness observed by family members upon awakening. Initially unresponsive, the patient subsequently regained consciousness, followed by a postictal state lasting approximately 30 minutes, characterized by mental confusion. Suspecting hypoglycemia, oral feeding was attempted, resulting in transient improvement. However, a subsequent episode occurred, characterized by urinary incontinence without involuntary movements. Blood was noted in the oral cavity, suggestive of tongue biting. In the emergency department, the patient was dehydrated, presented with confused and disoriented speech, and had hypertension (blood pressure of 195/98 mmHg and heart rate of 78 beats per minute). He was afebrile, normoglycemic, and exhibited no acute neurological deficits. Cranial computed tomography (CT) without contrast revealed a well-defined and rounded acute hyperdensity in the temporal horn of the left lateral ventricle (Figure 1) suggestive of hemorrhage, surrounded by a faint halo of perilesional edema, along with signs of reabsorption of the intraventricular hemorrhage in the frontal horn of the left lateral ventricle visualized seven years earlier, and unchanged chronic microvascular sequelae, evidenced by lacunar lesions in the basal ganglia and radiated crowns in the left hemisphere and right cerebellar hemisphere. Laboratory tests were unremarkable except for blood urea nitrogen of 79 mg/dL and serum creatinine of 2.64 mg/dL (in concordance with the preexisting chronic kidney disease). The electroencephalography revealed sparse epileptiform activity in the left medial temporal region. He was treated with sodium valproate, with no evidence of further seizures. 

**Figure 2:**
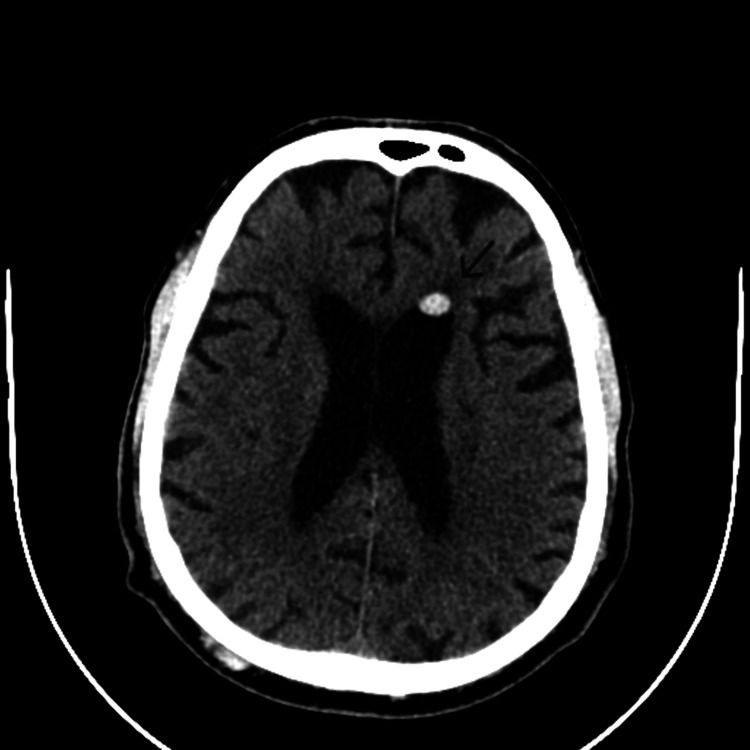
Follow-up non-contrast-enhanced brain CT showing new hyperdense focus in the frontal horn of the same ventricle (arrow)

Due to the suspicion of intracranial hemorrhage, antiplatelet therapy, which the patient had been taking chronically for ischemic heart disease, was discontinued. On follow-up CT, resorption of the hyperdensity in the temporal horn of the left lateral ventricle was documented, but a new hyperdense focus was observed in the frontal horn of the same ventricle (Figure 2), in a location matching that seen in imaging studies performed eight years earlier. This focus exhibited a similar morphology and was absent on the admission CT. At this point, a retrospective comparative analysis was performed by Neuroradiology using all prior cranial CT and MRI studies, revealing a fluctuating temporal evolution of the lesion. The lesion was observed to migrate from the left frontal horn to the ipsilateral temporal horn. Considering the patient’s history of vitrectomy performed several years earlier and the absence of such findings in imaging studies conducted prior to this procedure, it was concluded that it represented the migration of silicone oil particles from the vitreous humor of the eye to the intraventricular space, with mobilization according to the patient’s head movements. Once the CT imaging ruled out intraventricular hemorrhage, antiplatelet therapy was resumed. The patient remained asymptomatic throughout the hospital stay and during follow-up.

## Discussion

Since the onset of the use of silicone oil, only a limited number of reports have described the migration of this substance to the cerebral ventricular system [5,7]. On imaging, these findings can be mistaken for intracerebral hemorrhage or neoplasms, potentially leading to significant implications for patient management.

The mechanism underlying the diffusion of silicone oil and its consequent accumulation in specific intracranial sites remains unclear. Various pathophysiological mechanisms have been suggested, such as the emulsification of silicone oil and elevated intraocular pressure, which may facilitate the migration of silicone droplets into the optic nerve [8]. It is hypothesized that silicone oil enters the brain through the lamina cribrosa, a mesh-like structure at the optic nerve head that serves as a pressure barrier while permitting nerve fibers to exit the eye and form the optic nerve [9]. Congenital ocular abnormalities, such as optic pits or colobomas, may also contribute to this process. Additionally, pre-existing conditions like glaucoma or damage to the optic nerve and optic disc may increase the likelihood of silicone oil migration [10].

Most patients remain asymptomatic, with the position of the silicone oil in the ventricles often shifting in response to changes in head position. Nearly all reported cases have been asymptomatic and did not necessitate intervention [5,7]. However, migration of silicone oil to the third and fourth ventricles can obstruct cerebrospinal fluid outflow, potentially leading to hydrocephalus [11].

Previous studies have outlined several methods for differentiating intracranial silicone oil from intracranial hemorrhage on CT, including the higher attenuation of silicone oil and its persistent anti-dependent positioning [12,13]. In some cases, the migration pattern can be noticed through the presence of this substance in the optic globe and optic nerve. This distinction can ultimately avoid unnecessary reversal of antiplatelet or anticoagulation medications, ICU admission, or neurological procedures. Currently, there are no recommendations to modify the use of silicone oil based solely on the extremely low risk of intracranial migration.

## Conclusions

The aim of this report was to describe a rare occurrence of intraocular silicone oil migration into the cerebral ventricles. This phenomenon, although uncommon, poses significant diagnostic challenges and may be misinterpreted as intraventricular hemorrhage, leading to potential mismanagement of anticoagulation or antiplatelet therapy. Imaging studies, particularly CT and MRI, along with careful evaluation of a patient’s medical history, are crucial for accurate diagnosis. This case highlights the need for heightened awareness of silicone oil migration as a differential diagnosis, particularly in patients with a history of vitrectomy. Moreover, the case underscores the importance of interdisciplinary collaboration to ensure accurate diagnosis and appropriate treatment. Further studies are needed to better understand the pathophysiological mechanisms underlying silicone oil migration and its implications for patient management, as well as to establish clearer recommendations for differentiating silicone oil from intracranial hemorrhage in clinical practice.
